# Seroprevalence and associated risk factors of dromedary camel (*Camelus dromedaries) Toxoplasma gondii* in selected districts of Borana zone, Oromia, Ethiopia (short communication)

**DOI:** 10.1186/s12917-025-04908-4

**Published:** 2025-07-05

**Authors:** Selenat Getachew, Kubsa Wegene, Haben Fesseha Gebremeskel, Isayas Asefa Kebede

**Affiliations:** 1 School of Veterinary Medicine, Wolaita Sodo University, P. O. Box 138, Wolaita Sodo, Ethiopia; 2 Government Yabello District Veterinary Clinic Health Expert, Borana, Ethiopia; 3 School of Veterinary Medicine, Ambo University, P. O. Box 19, Guder, Ethiopia

**Keywords:** Camel, *Toxoplasma gondii*, Latex agglutination test, Borana

## Abstract

**Background:**

Toxoplasmosis in camels is an important zoonotic infection with considerable economic and public health impacts, particularly in the pastoral regions of Ethiopia.

**Methods:**

A cross-sectional study was conducted in selected districts of the Borana zone, Southern Oromia of Ethiopia, to estimate the *Toxoplasma gondii* seroprevalence infection and associated risk factors in camels. Accordingly, 352 camel blood samples were randomly collected. Then, the sera were separated from the blood and analyzed using a latex agglutination test.

**Results:**

According to this study, the seroprevalence of camel *T. gondii* in the study districts using the latex agglutination test was 7.9%. The current study revealed that the *T. gondii* seroprevalence was relatively higher in the Miyo (9.1%) district. The males (8.9%), the adult age group (5.8%), and the poor-condition camels (21.0%) had higher seroprevalence. There was a statistically significant association between the body condition and the seroprevalence of camel *T. gondii* (*p* = 0.016). However, no statistically significant difference was noted between the seroprevalence of camel *T. gondii* and the peasant association, districts, age, and sex.

**Conclusion:**

This study revealed a significant prevalence of *Toxoplasma gondii* infection in camels, posing a potential public health risk in the study area. To effectively control the disease and minimize its impact on both animal and human health, it is important to raise public awareness, implement practical biosecurity measures, and conduct further comprehensive studies.

## Background

In developing nations, between 50 and 100 million people rely on the pastoral economy for their subsistence; 60% of these individuals reside in over 21 African nations, primarily in their driest regions [1–3]. Ethiopian pastoralists, who move seasonally from one location to another in search of water and grassland for their animals, rely almost entirely on livestock for their subsistence [2, 3]. The world’s camel population is thought to be around 35 million [3].

Most found in semi-arid and dry regions of the world, dromedary camels (*Camelus dromedaries*) are among the most significant livestock. Ethiopia, the third-most camel-populated country in the world, is thought to have 8 million dromedary camels, which are primarily found in the Southern, Eastern, and Northeast pastoral regions [4]. Afar (34%), Somali (42%), and Borana (24%) are the principal ethnic groups in Ethiopia that possess camels [5]. Borana pastoralists are nomadic people who live in southern Ethiopia and northern Kenya. They kept livestock as their major source of income and moved periodically in search of food and water. Savannah vegetation predominates throughout the rangelands of Borana, with various amounts of open grasslands, perennial herbaceous vegetation, and woody vegetation [6].

Camels were historically believed to be resistant to most livestock diseases. As more research was done, it was discovered that camels are susceptible to a wide range of pathogenic agents, including *Toxoplasma gondii*, *trypanosomes*, and others [7, 8]. Camel toxoplasmosis is a globally distributed disease of significant economic and public health concern, with a worldwide seroprevalence of 28.16% [9]. In Ethiopia, the prevalence varies by region: 49.62% in the Fentale district [10] and 8.33% in the Borana zone [11]. The disease contributes substantially to camel morbidity and mortality [3, 11].

*Toxoplasma gondii* is an obligate intracellular protozoan parasite with a wide geographic range, capable of infecting many species of birds and mammals. It has a facultative indirect life cycle. The definitive hosts of *T. gondii* are members of the Felidae family. It can be infected by consuming mature oocysts. Once infected, they shed sporulated oocysts into the environment. These oocysts can then be ingested by intermediate hosts, such as cattle, sheep, goats, chickens, pigs, and camels, which act as reservoirs and contribute to the transmission of the parasite to humans [10–13]. Humans typically become infected by accidentally ingesting oocysts through contaminated food or water, or by inhaling or ingesting them from contaminated soil or surfaces, especially those contaminated by infected cats or wild felids [12, 13]. *Toxoplasma gondii* infection in camels has been associated with clinical signs such as abortion, along with pathological findings like hemorrhagic enterocolitis, toxoplasmic peritonitis, and reproductive issues, highlighting the zoonotic risk and the need for preventive measures [14].

The *T. gondii* can be transmitted to humans, particularly, through the consumption of milk and edible tissues of carrier camels [10, 15]. Women, primarily responsible for animal care, are particularly vulnerable to *T. gondii* infection [16]. This is of particular concern in pastoral communities, where raw milk and, to a lesser extent, raw meat are commonly consumed [17]. Serological tests, including direct agglutination test (DAT), Modified Agglutination Test (MAT), and latex agglutination test (LAT), are both reliable and efficient for detecting *T. gondii* infection in humans and animals, viz., camels, cattle, sheep, and goats [18]. These tests offer a reliable method for detecting anti-*Toxoplasma gondii* antibodies in animals [18–20].

Despite these advancements in diagnostic methods, data on the seroprevalence of *T. gondii* in both humans and animals in Ethiopia remain limited, even though consumption of raw or undercooked meat has been linked to human toxoplasmosis [11]. Some studies have reported camel toxoplasmosis in Ethiopia, particularly in agro-pastoral regions, viz. Fentale district and the Central Afar Region, where prevalence rates of 49.62% and 68.2% were found using the direct agglutination test (DAT) [10] and the Modified Agglutination Test (MAT) [21], respectively. However, there is a lack of comprehensive data from the study districts, where toxoplasmosis continues to pose a significant public health and economic issue [16].

The Borana zone’s pastoral lifestyle, characterized by the frequent consumption of raw meat and milk, close contact with cats and dogs, and reliance on unimproved water sources, significantly heightens the risk of *T. gondii* infection [10]. This study focuses on the Gomole and Miyo districts of the Borana zone, where pastoralists rely heavily on camels, suggesting an increased risk of toxoplasmosis. The status of camel toxoplasmosis in this area is sparse, necessitating an updated investigation. The Borana zone’s (including study areas) unique dietary habits and pastoral practices require a tailored epidemiological assessment. Up-to-date data are crucial for informing public health interventions and education programs to mitigate toxoplasmosis risk among pastoralists. This research will fill a significant gap by providing contemporary data on camel toxoplasmosis in a pastoralist setting.

Therefore, this study aims to estimate the seroprevalence of *Toxoplasma gondii* infection and identify associated risk factors in selected districts of the Borana zone, Southern Oromia, Ethiopia.

## Results

### Seroprevalence of *Toxoplasma gondii*

Out of 352 camels tested for *T. gondii* using the LAT, the overall seroprevalence was found to be 7.9% (95% CI: 5.5–11.3) in the study areas. Similarly, Miyo district had a higher seroprevalence of 9.1% (95% CI: 5.6–14.4) compared to the Gomole district (Table 1).

### Factors associated with the seroprevalence of *Toxoplasma gondii*

The univariable logistic regression analysis revealed that age and body condition score (BCS) were statistically significant risk factors associated with *T. gondii* infection in the study areas (age: *p* = 0.049, BCS: *p* = 0.019). Older camels had a threefold higher risk of *T. gondii* infection than adult camels (AOR: 3.3; 95% CI: 1.0-11.1). Similarly, camels with poor BCS were four times more likely to be infected compared to those with good BCS (AOR: 3.7; 95% CI: 1.2–11.1) (Table 1).

**Table 1:** Univariable logistic regression analysis of risk factors for seroprevalence of camels *T. gondii*

Variables	Category	NAE	LAT Positive	%	95%CI for %	AOR	95%CI for AOR	*p*-value
**District**	Gomole	176	12	6.8	3.9–11.6	Ref	-	-
	Miyo	176	16	9.1	5.6–14.4	1.4	0.6-3.0	0.432
**PAs**	Ola	56	2	3.6	0.9–13.4	Ref	-	-
	Kela kufa	60	3	5.0	1.6–14.6	1.4	0.2–8.8	0.706
	Buya	56	3	5.4	1.7–15.5	1.5	0.3–9.5	0.649
	Gomo maca	60	5	8.3	3.5–18.7	2.5	0.5–13.2	0.295
	Bildin	60	6	10.0	4.5–20.7	3	0.6–15.5	0.190
	Tumtu	60	9	15.0	7.9–26.5	4.8	0.9–23.1	0.053
**Age**	Young	66	4	6.1	2.3–15.2	Ref	-	-
	Adult	224	13	5.8	3.4–9.8	0.9	0.3-3.0	0.938
	Old	62	11	17.7	10.0-29.4	3.3	1.0-11.1	0.049
**Sex**	Female	117	7	5.9	2.7–12.1	Ref	-	-
	Male	235	21	8.9	5.9–13.4	1.5	0.6–3.7	0.338
**BCS**	Good	75	5	6.7	2.8–15.2	Ref	-	-
	Moderate	215	10	4.7	2.5–8.4	0.7	0.2–2.1	0.500
	Poor	62	13	21.0	12.5–32.9	3.7	1.2–11.1	0.019
**Total**		**352**	**28**	**7.9**	**5.5–11.3**			

NB: NAE: Number of Animals Examined, %: Prevalence, LAT: Latex Agglutination Test, AOR: Adjusted Odd Ratio

Potential risk factors with a p-value ≤ 0.25 in the univariable analysis were included in the multivariable logistic regression model. However, in the multivariable analysis, only BCS remained significantly associated with *T. gondii* infection (*p* < 0.05). Camels with poor BCS were three times more likely to be infected compared to those with good BCS (AOR: 3.3; 95% CI: 1.0–10.5). We tested the model’s fit using the Hosmer-Lemeshow test (HLχ2 = 161.36; Prob > χ2 = 0.0420) and the ROC curve (AUC = 0.68), which indicated acceptable performance (Table 2).

**Table 2:** Multivariable logistic regression analysis of risk factors for seroprevalence of *T. gondii in camels*

Variables	Category	%	AOR	95%CI for AOR	*p*-value
**BCS**	Good	6.7	Ref	-	-
	Moderate	4.7	0.6	0.2–1.8	0.360
	Poor	21.0	3.3	1.0-10.5	0.016

NB: %: Prevalence, AOR: Adjusted Odd Ratio

## Discussion

The present study revealed an overall seroprevalence of *T. gondii* in camels of 7.9% using the LAT. This finding is consistent with the reports by Kale [22], who found a prevalence of 6.3% in the Bandari region of Somalia using LAT.

Previous studies in Ethiopia have reported *T. gondii* seroprevalence rates ranging from 8.3 to 68.2%, depending on the region and diagnostic method used [10, 11, 21]. Similarly, Selim et al. [23] reported a seroprevalence of 46.9% for *T. gondii* using ELISA in three regions of Egypt. Khattab et al. [24] found an even higher rate of 64.51% with the same method.

Differences in climatic and management conditions may have contributed to variations in *T. gondii* seroprevalence in camels. Temperature and humidity, particularly warmer and more humid climates, affect the survival and transmission of *T. gondii* oocysts, favoring their persistence [11]. Differences in management practices, including veterinary care and culling programs, also impacted camel susceptibility to infections like *T. gondii* [22, 23].

Additionally, the community has a tradition of supplementing camel diets with salt to promote weight gain and protect against illnesses, including gastrointestinal parasitic infections and respiratory disorders. Table salt has been reported to inactivate *T. gondii*, indicating its potential role in mitigating the risk of transmission [25, 26]. Research has shown that salt curing can effectively inactivate *T. gondii* tissue cysts, with the degree of inactivation depending on factors such as maturation time, temperature, and salt concentration [27]. Given that camels are uniquely adapted to tolerate high salinity levels due to their renal adaptations [28–30], it is plausible that salt supplementation could contribute to the relatively low *T. gondii* seroprevalence observed in the district. However, the specific effectiveness of salt in reducing *T. gondii* transmission in camels remains underexplored. Thus, further research is required to conclusively determine the impact of salt supplementation on *T. gondii* transmission in camels. This practice, along with other factors, may help explain the observed low seroprevalence of *T. gondii* in the study area.

In this study, no statistically significant difference (*p* > 0.05) in seroprevalence was found between different sexes (male: 8.9% vs. female: 5.9%). Similarly, Khamesipour et al. [31] and Gebremedhin et al. [10] also reported that sex was not a risk factor for *T. gondii* prevalence. In contrast, Selim et al. [23] observed a significant association between sex and *T. gondii* seroprevalence, reporting that females were more susceptible to infection than males.

Gebremedhin et al. [11] also found that female camels had 3.5 times higher odds of testing positive for *T. gondii* compared to males, attributing this difference to the stress of pregnancy and lactation, which can affect their physiological and immunological states. Additionally, their study showed that the seroprevalence of *T. gondii* increased with age, with older camels (> 8 years) having significantly higher seroprevalence than younger ones (≤ 4 years). In line with this, the current study also observed a higher seroprevalence of *T. gondii* in older camels (17.7%) compared to younger ones (6.1%), consistent with the findings of Selim et al. [23]. Older camels are more likely to have encountered risk factors for *T. gondii* infection, suggesting that the probability of infection increases with age due to prolonged exposure [23].

This study, conducted in the Miyo and Gomole districts, found no significant association between *T. gondii* infection and the variables examined, including study districts and peasant associations. These results align with [11], who also found no significant relationship between these factors and infection (*p* > 0.05). The lack of association may be due to other factors such as camel population, management practices, age, elevation, and precipitation. However, other scholars reported a significant association (*p* < 0.05) between disease prevalence and specific pastoral associations in a different context, suggesting that unmeasured risk factors may influence seroprevalence [10]. Although variation was observed across districts and peasant associations, as well as age and sex, these differences were not statistically significant. Further studies with larger sample sizes may help explore local variation more clearly.

The current study found a significant association (*p* < 0.05) between BCS and the seroprevalence of *T. gondii*. The results showed that camels in poor condition had a significantly higher seroprevalence of *T. gondii* (21.0%) compared to those in good condition (6.7%) (*p* < 0.05). Camels with poor BCS were three times more likely to be infected than those with good BCS (AOR: 3.3; 95% CI: 1.0–10.5). This finding is consistent with the results of Hadush et al. [21], who reported that poor body condition and lower disease resistance, linked to the immune system, contributed to a high seroprevalence (74.6%) in camels with poor BCS.

Due to constraints in manpower and funding, the study was unable to assess confounding factors such as management practices, contact with felines (the primary hosts of *T. gondii*), and water contamination risks. We did not test environmental samples or local feline populations, which are important sources of *T. gondii* oocysts. We also did not include human serological data, which limits our ability to assess zoonotic transmission in this One Health context. These factors might have influenced the results, and future research should address them for a more comprehensive analysis. Additionally, the **LAT** used in this study has limitations, including lower sensitivity and specificity compared to **ELISA**, which can result in false positives and negatives. As a semi-quantitative test, **LAT** cannot provide precise antibody measurements. While **LAT** is cost-effective and rapid, it may not be reliable in populations with low antibody titers or chronic infections, and cross-reactivity with other pathogens could affect its accuracy. In contrast, **ELISA** offers greater sensitivity and specificity but requires more resources, time, and specialized equipment. However, our study aims to provide valuable insights for livestock and policy stakeholders, emphasizing the need for further, more comprehensive research on *T. gondii* infections, especially as the pastoral community continues to rely on camels for their livelihoods.

## Conclusion

The current study demonstrated that camels in the Borana zone have a significantly lower prevalence of *T. gondii* infection. Among the associated risk factors examined, BCS was the only significant risk factor associated with *T. gondii* seropositivity. To improve camels’ body condition, the introduction of higher-quality feed should be encouraged. Furthermore, preventive measures should prioritize educating camel herders and owners about the risk factors associated with the *T. gondii* infection, including the consumption of raw or undercooked meat and the proper management of edible offal.

## Materials and methods

### Study area

**Fig. 1:**
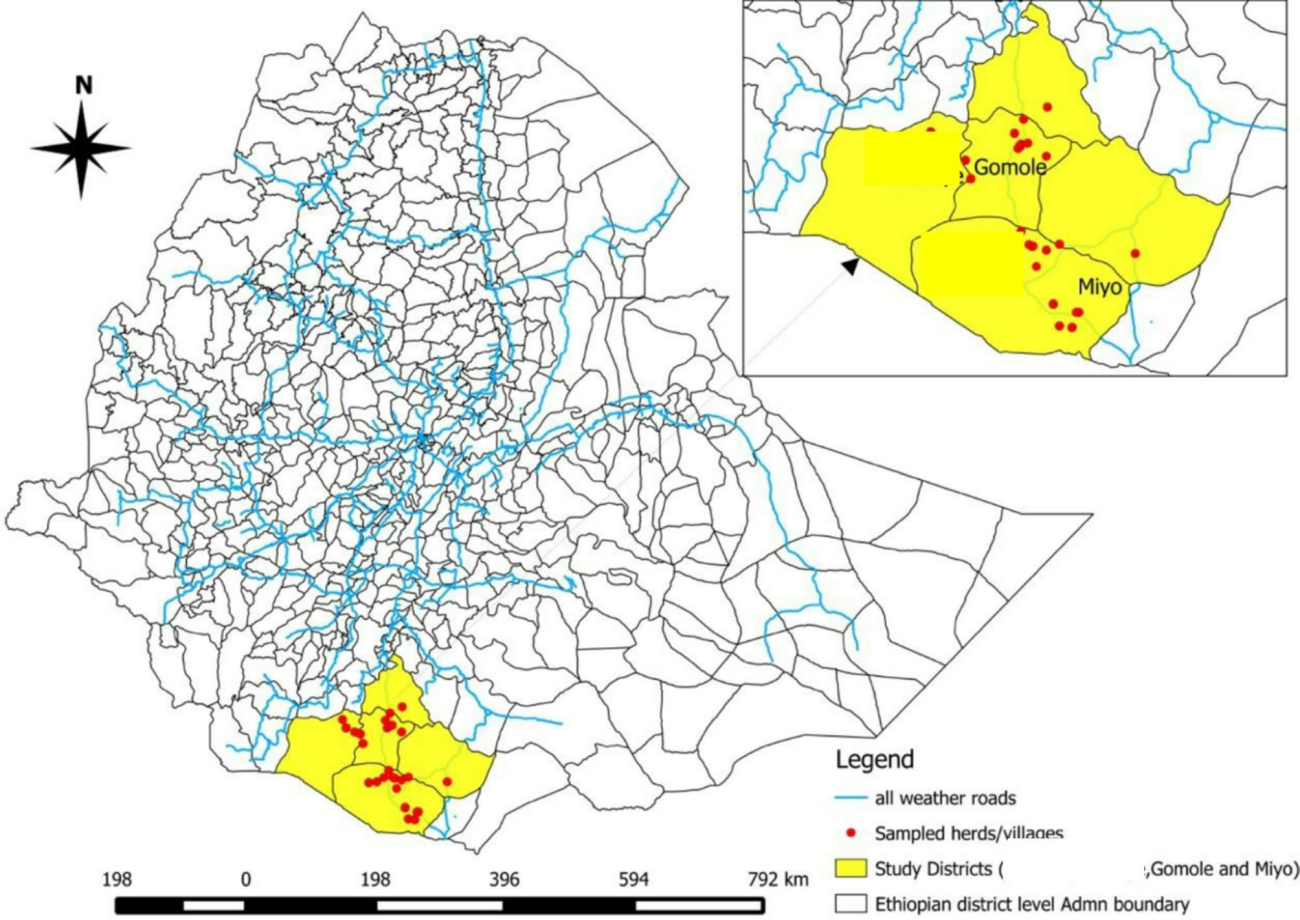
Map of the study sites (ArcGIS, 2018)

The study was conducted in two selected districts (Gomole and Miyo) of the Borana zone of Oromia Regional State, southern Ethiopia (Fig. 1). The zone is in the Southern part of the state between 3°36 − 6°38’North latitude and 3°43’- 39°30’ East longitude. The capital city of the Borana zone is Yabello, which is 575 km away from the capital city, Addis Ababa, in the south direction. The zone has a latitude range between 943 and 2,400 m above sea level with an average annual rainfall of 300 to 900 mm, exhibiting a bimodal rainfall (long and short rainy seasons). The annual temperature varies between 19 and 35^0^ C. The zone has an estimated population of 962,489 (male 487,024 and female 475,465), with 91.2% of the population living in rural areas [32]. The zone has about 1,844,027 cattle, 1,299,451 goats, 664,307 sheep, 216,131 camels, 414,021 poultry, 114,952 donkeys, 2,624 horses, and 20,807 mules [33]. In the study area, camels were raised using an extensive system of production alongside other livestock, such as small ruminants and cattle [34].

### Study animals

The study animals included camels (*Camelus dromedarius*) managed in an extensive production system, as well as indigenous breeds of both sexes and different age groups, all older than six months, including pregnant and lactating camels.

### Study design

A cross-sectional study was carried out between October 2017 and June 2018 to determine the seroprevalence of camel *T. gondii* in two purposively selected districts (Gomole and Miyo) in the Borana zone. In addition to being suitable in terms of accessibility, camel population, and the availability of infrastructure, the study districts were purposefully chosen from among the total of 13 districts in the Borana zone to represent the midland and lowland agroecology.

### Sample size determination and sampling techniques

The required sample size for the study was calculated using the Thrusfield formula [35]:

$$N = \frac{{{Z^2} \times {P_{exp}}\left( {1 - {P_{exp}}} \right)}}{{{d^2}}} $$

= 1.962 × 0.0833 (1-0.0833)/0.052 = 3.84 × 0.076/0.0025 = 117.3

Where N is the number of required samples, Z = 1.96 for a 95% confidence interval, Pexp is the predicted prevalence (8.33%) [11], and d is the desired absolute precision (5%). Accordingly, the minimum sample size [N] was 118 camels. However, a total of 352 animals were included in this study to increase precision and representativeness.

In this study, camel herds were purposively selected based on infrastructure accessibility and the willingness of the owners. Individual animals were then chosen from these herds using systematic random sampling methods. Briefly, camels were selected from herds whose owners gave consent and were available at the time of sampling. In each herd, animals were chosen using a systematic approach (every fourth camel) when possible. In cases where systematic selection was not practical, animals were selected based on the owner’s presentation. This may have introduced some selection bias, which we acknowledged as a limitation.

### Sample collection, transportation, and serological analysis

The 352 camels were restrained with assistance from their owners, and 5–10 ml of blood was drawn from the jugular vein using a disposable plain vacutainer tube and needle. The blood samples were then transported to the Yabello regional veterinary laboratory, where they were left overnight to coagulate. Afterward, the samples were separated and decanted into serum tubes (cryovial tubes) and stored at -20 °C before undergoing a latex agglutination test (LAT). Each sample was labeled with a unique code, and information on the camels, including age (young: ≤ 4 years, adult: 4–8 years, and old: > 8 years) [11], body condition score (BCS) (poor, medium, and good), and sex (female or male), was recorded in a separate case book. A camel in poor body condition has visible ribs and prominent bones, indicating a lack of fat reserves, while a camel in good condition has well-covered ribs with no visible bones, and a camel in medium condition shows slightly visible ribs with a balanced fat covering [36]. The site, species, and sex were also documented on the vacutainer and serum tubes (ethical approval protocol: RN-WSU 41/22/2241).

### Latex agglutination test (LAT)

All serum samples were tested for IgG antibodies against *T. gondii* using a commercial LAT kit (Toxo screen DA, biomerieux^®^, France) [37] according to the manufacturer’s instructions. The serum samples and *T. gondii* antigen were left at room temperature for 30 min before the test. To perform the LAT, 50 µl of each serum sample was placed on the test plate. The antigen vial was then opened carefully, and 25 µl of antigen was added next to each serum sample. Using a stirrer, the serum and antigen were mixed and spread across the entire circle of the plate. After manually rotating the plate for 4 min, the results were read immediately [38]. If the antigen settled at the bottom of the well and no agglutination was observed, it was recorded as negative. If clear agglutination occurred above half of the well at any dilution, it was considered a positive result. The sensitivity and specificity for the kit are 96.22 and 98.80%, respectively [16].

### Data management and analysis

All data from the study were recorded in Microsoft Excel 2016 and analyzed using STATA (version 14.0) statistical software (Stata Corp., College Station, USA). Serorevalence of *T. gondii* was calculated, and logistic regression was used to assess the relationship between prevalence and the hypothesized risk factors, namely sex, age, body condition score, and study sites (Peasant Associations: PAs). In the univariable analysis, explanatory variables with a p-value ≤ 0.25 (maximum likelihood ratio test) were selected for inclusion in the multiple logistic regression analysis. Final models were constructed manually using a forward stepwise selection approach. Confounders were identified as variables that altered the coefficient of significant variables by more than 25%. Kruskal gamma statistics were used to assess multicollinearity between predictors, with variables having gamma values between − 0.6 and + 0.6 included in the multivariate logistic regression model. The final model was used to calculate the odds ratios (OR) and 95% confidence intervals (CI) for factors influencing the outcome variables. Statistical significance was set at a p-value < 0.05. Hosmer and Lemeshow statistics, as well as the Receiver Operating Curve (ROC), were used to assess model fit and validity [39].

## Data Availability

All data generated or analyzed during this study are included in this published article.

## Abbreviations

CSA: Central Statistical Agency
LAT: Latex Agglutination Test
USAID: United States Agency for International Development
UNDP: United Nations Development Program
